# Extracorporeal shockwave against inflammation mediated by GPR120 receptor in cyclophosphamide-induced rat cystitis model

**DOI:** 10.1186/s10020-018-0062-1

**Published:** 2018-11-27

**Authors:** Yi-Ling Chen, Yuan-Ping Lin, Cheuk-Kwan Sun, Tien-Hung Huang, Hon-Kan Yip, Yen-Ta Chen

**Affiliations:** 1 Division of Cardiology, Department of Internal Medicine, Kaohsiung Chang Gung Memorial Hospital and Chang Gung University College of Medicine, No. 123, Dapi Road, Niaosong District, Kaohsiung, 83301 Taiwan; 2 Department of health and Beauty, Shu-Zen Junior College of Medicine and Management, No.452, Huanqiu Rd. Luzhu Dist., Kaohsiung, 82144 Taiwan; 3 Department of Emergency Medicine, E-Da Hospital, I-Shou University School of Medicine for International Students, No. 1, Yi-Da Road, Jiao-Su Village, Yan-Chao District, Kaohsiung, 82445 Taiwan; 4 Institute for Translational Research in Biomedicine, Kaohsiung Chang Gung Memorial Hospital, Kaohsiung, 83301 Taiwan; 5 Center for Shockwave Medicine and Tissue Engineering, Kaohsiung Chang Gung Memorial Hospital, Kaohsiung, 83301 Taiwan; 6 Department of Medical Research, China Medical University Hospital, China Medical University, Taichung, 40402 Taiwan; 7 Department of Nursing, Asia University, Taichung, 41354 Taiwan; 8 Division of Urology, Department of Surgery, Kaohsiung Chang Gung Memorial Hospital, No. 123, Dapi Road, Niaosong District, Kaohsiung, 83301 Taiwan

**Keywords:** Extracorporeal shockwave treatment, GPR120, Interstitial cystitis, Inflammation

## Abstract

**Background:**

We tested the hypothesis that extracorporeal shockwave treatment (ESWT) can abolish inflammation and restore urothelial barrier integrity in acute interstitial cystitis by upregulating the fatty acid receptor GPR120.

**Methods:**

A total of 30 female Sprague-Dawley rats were categorized into five groups: (1) sham-operated rats (SC); (2) rats treated with ESWT (SC + ESWT); (3) rats with bladder irritation using 150 mg/kg cyclophosphamide through intraperitoneal injection; (4) cyclophosphamide rats treated with ESWT (cyclophosphamide+ESWT); (5) cyclophosphamide rats treated with GPR120 agonist (cyclophosphamide+GW9508).

**Results:**

On Day 3, urine and bladder specimens were collected for biochemical, histopathological, immunological, and immunoblotting analysis. Following stimulation with cyclophosphamide, the inhibition of the elevated levels of TAK1/NF-κB and phospho-TAK1/NF-κB by ESWT and GPR120 agonists in RT4 cells was associated with a suppression of NF-κB translocation from the cytosol to the nucleus. Accordingly, this anti-inflammatory effect was abolished by GPR120 antagonist and knockdown of GPR120. Histologically, bladder inflammation in cyclophosphamide-treated rats was suppressed by GW9508 or ESWT. Masson’s trichrome and Sirius red staining revealed that cyclophosphamide treatment enhanced synthesis of extracellular matrix in rats that was reversed by GW9508 or ESWT. Upregulated pro-inflammatory mediators and cytokines in the cyclophosphamide-treated rats were also suppressed in the GW9508- or ESWT-treated rats. The significantly increased inflammatory cell infiltration as well as the impaired urothelial integrity of the bladder after cyclophosphamide treatment were reversed by treatment with GW9508 or ESWT.

**Conclusions:**

These findings suggest that GPR120, the sensing receptor for ESWT, may be useful in the treatment of interstitial cystitis by inhibiting inflammatory response in bladder cells.

## Introduction

Interstitial cystitis (IC) is a clinical syndrome characterized by urinary frequency, nocturia, and pelvic pain with unknown etiology. IC has a female predominance with an average age of diagnosis between 42 and 46 years (Patnaik et al. 2017). Although the etiology of IC is unknown, numerous theories defining the pathology of IC have been proposed, including altered barrier lining, afferent and/or central nervous system abnormalities, possible contribution of inflammatory or bacterial infection and abnormal urothelial signaling (Wang et al. 2016; Lazzeri et al. 2016; Regauer 2016; Gonzalez et al. 2014). Clinically, there is still no simple treatment that can eliminate the signs and symptoms of IC.

G protein-coupled receptors (GPCRs) constitute a family of seven transmembrane proteins that mediate many cellular processes. GPR120/free fatty acid receptor 4, a receptor from this family that is activated by fatty acids, has received much attention recently (Oh et al. 2010; Karakula-Juchnowicz et al. 2017; Fredriksson et al. 2003). Numerous studies have reported that GPR120 is ubiquitously expressed throughout the body, localized in different cell types, and found to regulate various physiological processes, including gut hormone secretion, islet function, osteoclastogenesis, anti-inflammation, and adipogenesis (Tanaka et al. 2008; Cornall et al. 2011; Hirasawa et al. 2005; Gotoh et al. 2007). Being a receptor of omega-3 fatty acids, GPR120 exerts its physiological effects through one of two pathways that involves either Gαq or β-arrestin-2. While the former is responsible for adipogenesis, the latter has been found to be anti-inflammatory (Oh and Walenta 2014). Interestingly, the stimulation of GPR120 with omega-3 fatty acids (ω-3 FAs) results in β-arrestin-2 coupling and causes the release of TGF-β activated kinase 1 (TAK1) from TAK1 binding protein (TAB1), thereby leading to the inactivation of TAK1 and abrogation of inflammatory cascades of the NF-κB and mitogen-activated protein kinases (MAPKs) pathways (Oh et al. 2010; Yin et al. 2016; Takaesu et al. 2003). Oh et al. have recently shown that the binding of ω-3 FAs to GPR120 exerts potent anti-inflammatory, anti-diabetic and insulin-sensitizing effects in macrophages and adipocytes. This anti-inflammatory property of GPR120 raises the possibility that targeting this receptor could have therapeutic potential against inflammatory diseases (Mo et al. 2013). Therefore, the present study aims at investigating the anti-inflammatory role of GPR120 in IC.

Since its first medical use for lithotripsy in 1980s, extracorporeal shock wave (ESWT) has been found to promote tissue repair and regeneration (Wang 2012). Although the exact mechanisms are still unclear, biological effects including direct stimulation of wound healing, neovascularization, and inhibition of painful sensations have been proposed (Yeh et al. 2012; Notarnicola and Moretti 2012; Sheu et al. 2015; Chen et al. 2015; Huang et al. 2016). We have recently demonstrated substantial amelioration of inflammation and oxidative stress in cyclophosphamide (CYP)-induced acute IC in a rat model after ESWT, but possible therapeutic mechanism remains to be elucidated (Chen et al. 2014a). The other aim of the current study is to investigate the role of ESWT in the anti-inflammatory effect of GPR120 in an experimental setting of IC.

## Materials and methods

### Chemicals and antibodies

GPR120 agonists GW9508 and docosahexaenoic acid (DHA) were obtained from Sigma-Aldrich (St. Louis, MO, USA); GPR120 antagonist AH7614 was from Tocris Bioscience (Ellisville, MO, USA). Anti-GPR120 antibody was purchased from Santa Cruz Biotechnology (Dallas, TX, USA); anti-Bax, anti-iNOS, anti-MCP-1, anti-NF-κB antibodies were from Abcam (Cambridge, UK). Anti-caspase 3, anit-IL-1β, anti-phospho-NF-κB, anti-PAPR, anti-phospho-γ-H2AX, anti-TAK1 (transforming growth factor-β-activated kinase 1), anti-phospho-TAK1, and anti-TNF-α antibodies were obtained from Cell Signaling Technology (Beverly, MA, USA) and anti-β-actin were from EMD Millipore (Danvers, MA, USA). Anti-NLRP3 and anti-IL-6 were form Protein Technologies (Tucson, AZ, USA) and Biorbyt Ltd. (Taipei, Taiwan), respectively.

### Cell culture

The urothelial cell line (RT4) was maintained in McCoy’s 5A medium (Thermo Fisher Scientific, Carlsbad, CA, USA) supplemented with 10% fetal bovine serum (Thermo Fisher Scientific), 1% penicillin, and 1% streptomycin (Thermo Fisher Scientific) and grown at 37 °C in a humidified atmosphere of 5% CO_2_. For experiments, confluent cells in cell culture flasks were trypsinized and seeded into plates at a cell density of 1 × 10^5^ cells/mL. At 60–70% confluency, cells were used for treatments. Cells were stimulated with GW9508 (100 μM), DHA (100 μM), AH7614 (100 μM), or ESWT for 4 h prior to CYP (10 μM) treatment for 24 h and then subjected to immunoblotting.

### siRNA silencing of GPR120

Small interfering RNA (siRNA) for rat GPR120 (Thermo Fisher Scientific) and scrambled siRNA were obtained commercially from Thermo Fisher Scientific. Transfection of RT4 cells (1 × 10^5^ cells per well in 6-well plate) was performed according to the manufacturer’s instructions. Before transfection, cells were treated with ESWT for 4 h. At the 48 h after transfection, total RNA and protein were extracted from the cells to quantify the relative expression level of GPR120 by RT-PCR and immunoblotting.

### RNA extraction and real-time RT-PCR

Total cellular RNA was isolated and prepared using RNeasy Mini Kit (Qiagen, Valencia, CA), following the manufacturer’s protocol. For quantitative real-time reverse transcriptase-PCR (qRT-PCR) experiment, cDNA was produced using the Applied Biosystems™ High-Capacity cDNA Reverse Transcription Kit with RNase Inhibitor (Thermo Fisher Scientific) and 50 ng of cDNA per sample was analyzed using an Applied Biosystems™ StepOne™ Real-Time PCR System together with gene-specific primers and TaqMan® Gene Expression Master Mix. Accompanying software was used for the acquisition of threshold cycle (Ct) values. Fold changes in expression quantities was calculated according to the 2^-ΔΔCT^ method.

### Animals

All procedures were approved by the Institute of Animal Care and Use Committee at Kaohsiung Chang Gung Memorial Hospital (IACUC no. 2014121816) and performed in accordance with the Guide for the Care and Use of Laboratory Animals by National Institutes of Health. Thirty female Sprague-Dawley (SD) rats (220 ± 30 g) provided by Charles River Technology, BioLASCO Taiwan Co., Ltd., Taiwan were randomized and equally categorized into five groups: (1) sham-operated rats (SC); (2) rats treated with extracorporeal shock wave (SC + ESWT); (3) rats with bladder irritation received one intraperitoneal injection of 150 mg/kg cyclophosphamide (CYP); (4) CYP rats treated with ESWT at energy of 0.15 mJ/mm^2^, 300 impulses (CYP + ESWT); (5) CYP rats treated by intravesical instillation with 5 μg/kg GPR120 synthetic agonist (CYP + GW9508). Focused ESWT was applied to the skin above the urinary bladder at 3 and 24 h after cyclophosphamide treatment. On the third day, overnight urine specimens were collected for ELISA before the rats were sacrificed. The bladders were then removed for histopathological, histoimmunological, and western analysis. An in vitro study was also performed in which urothelial RT4 cells were selectively treated with CYP, ESWT, GPR120 agonist/antagonist.

### Enzyme-linked immunosorbent assay, ELISA

The levels of IL-1β and IL-6 in urine were determined using commercially available ELISA kits from R&D Systems (Minneapolis, MN, USA) according to the manufacturer’s instructions.

### Histological analysis

Bladder specimens were fixed, embedded, sectioned, stained with hematoxylin and eosin (H&E), Masson’s trichrome, and Sirius red for light microscopy as we previously published (Chen et al. 2014a, b). The scoring system of H&E-stained bladder sections based on the degree of inflammatory cell infiltration, epithelial thinning, and mucosal distortion was as follows: 0 (none), 1 (≤10%), 2 (11–25%), 3 (26–45%), 4 (46–75%), and 5 (≥76%). Scoring was performed in 10 randomly chosen, non-overlapping fields (200×) for each animal. The mean value served as the final score.

Masson’s trichrome and Sirius red are the most frequently used stains for assessing collagen synthesis and deposition. The integrated area (μm^2^) of fibrosis on each section was calculated using a UTHSCSA ImageTool (IT) 3.0 (University of Texas Health Science Center, San Antonio). Three randomly selected HPFs (100×) were analyzed in each section. After determining the number of pixels in each fibrotic area per HPF, the numbers of pixels obtained from three HPFs were summated. The procedure was repeated in two other sections for each rat. The mean pixel number per HPF for each rat was then determined by summating all pixel numbers and dividing by 9. The mean integrated area (μm^2^) of fibrosis in bladder per HPF was obtained through dividing the mean pixel numbers by 19.24 (1 μm^2^ represented 19.24 pixels).

### Immunofluorescent examinations

The protocols for immunofluorescent (IF) examinations were also described previously (Chen et al. 2014a, b). Briefly, frozen sections incubated with primary antibodies specifically against CD68 (1:100; Abcam, Cambridge, UK) and ZO-1 (1:100, Abacm) at 4 °C overnight. Irrelevant antibodies were used as controls. Three sections of bladder specimens were analyzed in each rat. For quantification, three randomly selected HPFs (200× or 400× for IHC and IF studies) were analyzed in each section. The mean number per HPF for each animal was then determined by summation of all numbers divided by 9.

### Immunoblotting

Frozen tissues or cell samples were mechanically homogenized with 1× RIPA buffer (Cell Signaling Technology) containing 1× protease inhibitor cocktail (Roche, Indianapolis, IN). Proteins lysates were separated by SDS-PAGE on 7–12% acrylamide gradients. Proteins lysates were transferred to a nitrocellulose membrane (Bio-Rad Laboratories, Hercules, CA) or polyvinylidene difluoride membrane (Sigma-Aldrich) and probed with the monoclonal antibodies against Bax, caspase-3, GPR120, iNOS, IL-1β, IL-6, MCP-1, NLRP3, NF-κB, phospho-NF-κB, TAK1, phospho-TAK1, PARP, γ-H2AX, TNF-α. Immunoreactive bands were detected by ECL chemiluminescence (EMD Millipore) and quantified with Quantity One Image Software (Bio-Rad Laboratories).

### Statistical analysis

Data were expressed as mean ± SEM or mean ± SD. Results were analyzed by one-way analysis of variance (ANOVA), followed by Bonferroni’s post-hoc comparisons tests. Differences were considered significant when *p* < 0.05.

## Results

### ESWT increases GPR120 in a dose-dependent fashion

**Fig. 1:**
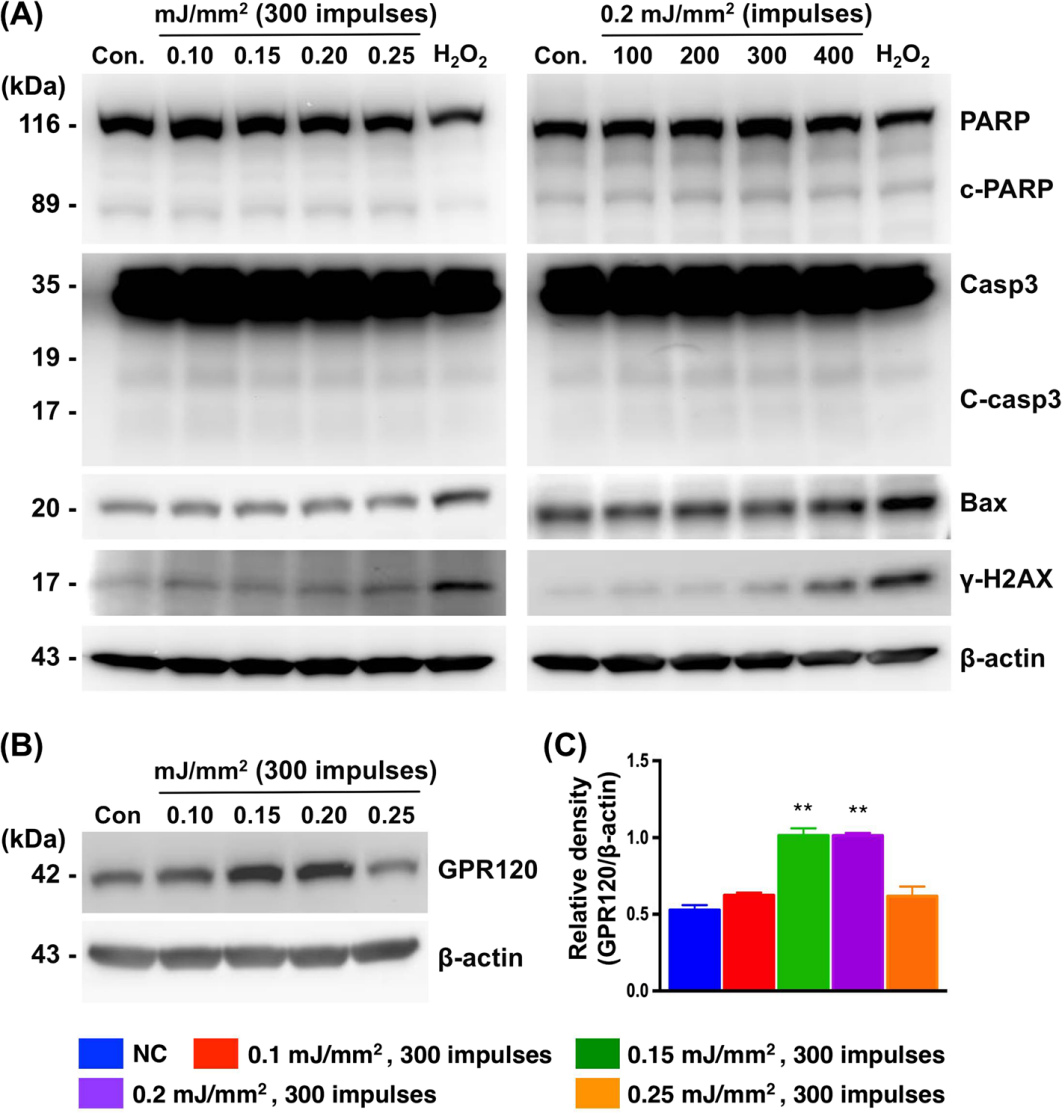
Upregulation of GPR120 by extracorporeal shock wave treatment (ESWT) in human urothelial RT4 cells. **a** Expressions of apoptosis-related proteins in RT4 28 h post-ESWT assessed by immunoblotting, including cleaved-PARP (c-PARP), cleaved-caspase 3 (c-casp 3), Bax, γ-H2AX. H_2_O_2_ (500 μmol/L) treatment used as positive control. **b** Optimal energy and frequency of ESWT for GPR120 upregulation. **c** Densitometric quantification of GPR120 in RT4 without (i.e., Con.) or with ESWT. β-actin used as internal control for immunoblotting. Values expressed as the mean ± SD of three independent experiments. *p < 0.05, **p < 0.01 vs. control; Significance of differences determined by one-way ANOVA followed by Bonferroni’s post-hoc comparisons tests

To determine whether RT4 cells displayed apoptosis in response to ESWT, we applied four different low-energy levels (0.10, 0.15, 0.20, and 0.25 mJ/mm^2^) and four different impulses (100, 200, 300, and 400) to determine the apoptosis and DNA damage protein expressions by immunoblotting after ESWT (Fig. 1a). H_2_O_2_-treated (500 μM) RT4 cells were used as positive control to verify the protein expressions of cellular apoptosis and DNA damage. All ESWT-treated RT4 cells maintained low expression levels of cleavage fragment of caspase 3, PARP, and Bax compared to those in the controls. However, increased γ-H2AX protein expression was observed at the level of 0.25 mJ/mm^2^ and 400 impulses. Optimal conditions for ESWT were found to be 0.15 mJ/mm^2^ and 300 impulses. Therefore, we chose the level of 0.15 mJ/mm^2^ with 300 impulses, 5 Hz for the following experiments. RT4 cells were treated with ESWT 300 impulses at an energy level of 0.10 to 0.25 mJ/mm^2^, and GPR120 expression was measured with immunoblotting after 24 h. The results showed that low energy (0.15 mJ/mm^2^) ESWT induced GPR120 and higher energy (0.25 mJ/mm^2^) decreased GPR120 expression after 24 h (Fig. 1b-c).

### ESWT enhanced GPR120-mediated anti-inflammatory effects

**Fig. 2:**
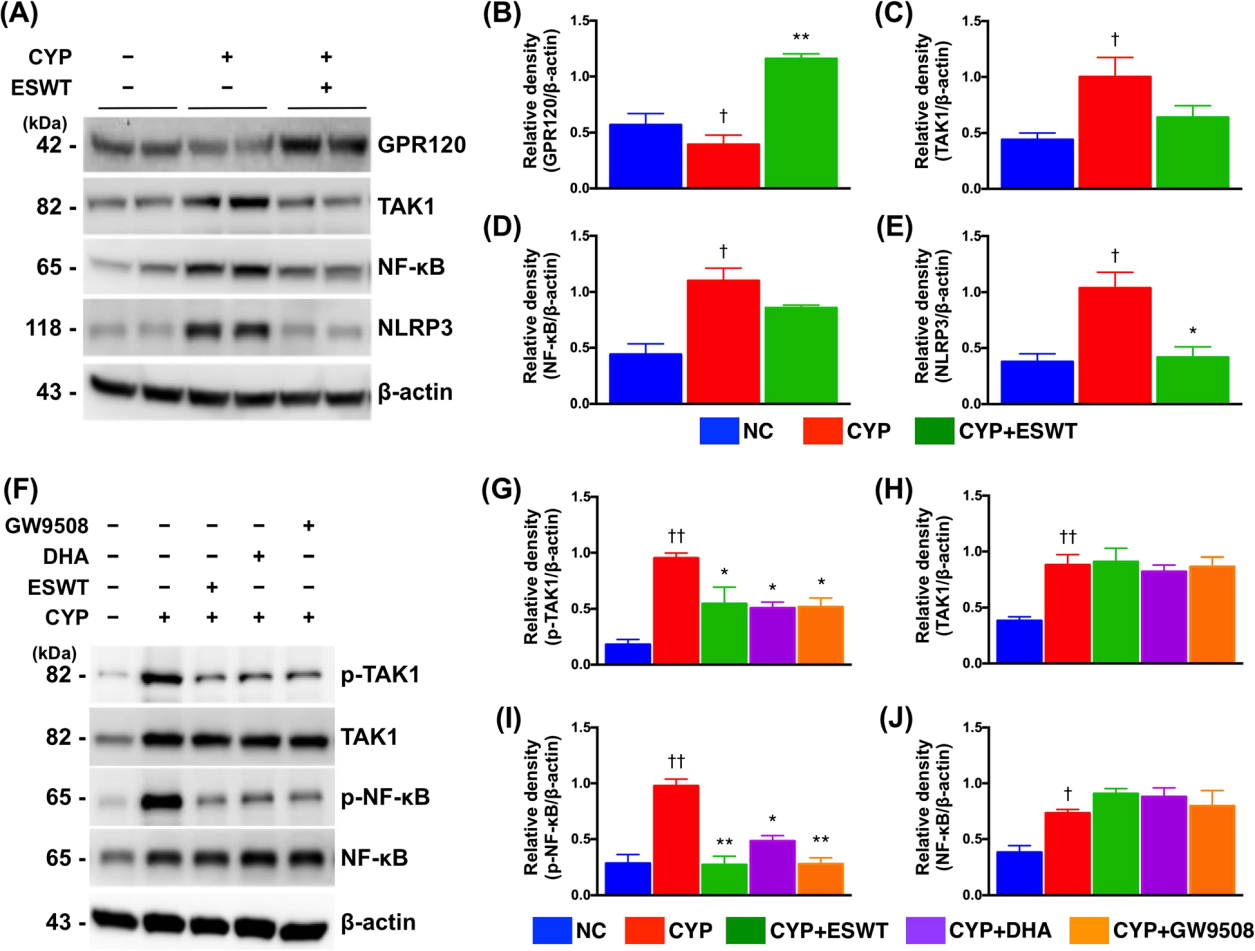
Effects of extracorporeal shock wave treatment (ESWT) and GPR120 agonists on cyclophosphamide (CYP)-stimulated inflammatory response in urothelial RT4 cells. **a-e** Protein expressions of pro-inflammatory (i.e., TAK1, NF-κB and NLRP3) and anti-inflammatory (i.e., GPR120) markers in CYP-treated RT4 cells at 24 h with and without prior ESWT. **f-j** Protein expressions of active pro-inflammatory markers (i.e., p-TAK1 and p-NF-κB) in CYP-stimulated RT4 cells at 4 h with and without prior treatment with GPR120 agonists (i.e., GW9508 and DHA) compared to those elicited through ESWT. β-actin used as internal control for immunoblotting. Values expressed as the mean ± SD of three independent experiments. *p < 0.05, **p < 0.01 vs. CYP; ^†^p < 0.05, ^††^p < 0.01 vs. control; Significance of differences determined by one-way ANOVA followed by Bonferroni’s post-hoc comparisons tests

Stimulation of GPR120 with a chemical/synthetic agonist GW9508 or a natural agonist ω-3 FAs docosahexaenoic acid (DHA) has been found to be anti-inflammatory in several cell types. To investigate the effect of ESWT on CYP-induced IC and its influence on total TAK1 and NF-κB protein expressions, CYP-treated RT4 cells treated with ESWT were allowed to settle for 24 h. The results showed significant increase in GPR120 expression and decrease in both TAK1 and NF-κB expressions compared to CYP-treated RT4 cells without ESWT (Fig. 2a-e). To study the changes in protein expressions of the phosphorylated (i.e., active) forms of TAK1 and NF-κB, the levels of both phosphorylated molecules were determined 4 hours after ESWT. The pattern of changes was found to be similar to that of the total forms at 24 h (Fig. 2f-j). To further understand whether the anti-inflammatory effect of GPR120 could be reproduced in the absence of ESWT, protein expressions of the phosphorylated forms of TAK1 and NF-κB in RT4 cells pretreated with GPR120-specific ligands (i.e., GW9508 and DHA) were determined at 4 h. The expressions of both phosphorylated molecules were found to decrease to levels comparable to those after ESWT (Fig. 2f-j).

**Fig. 3:**
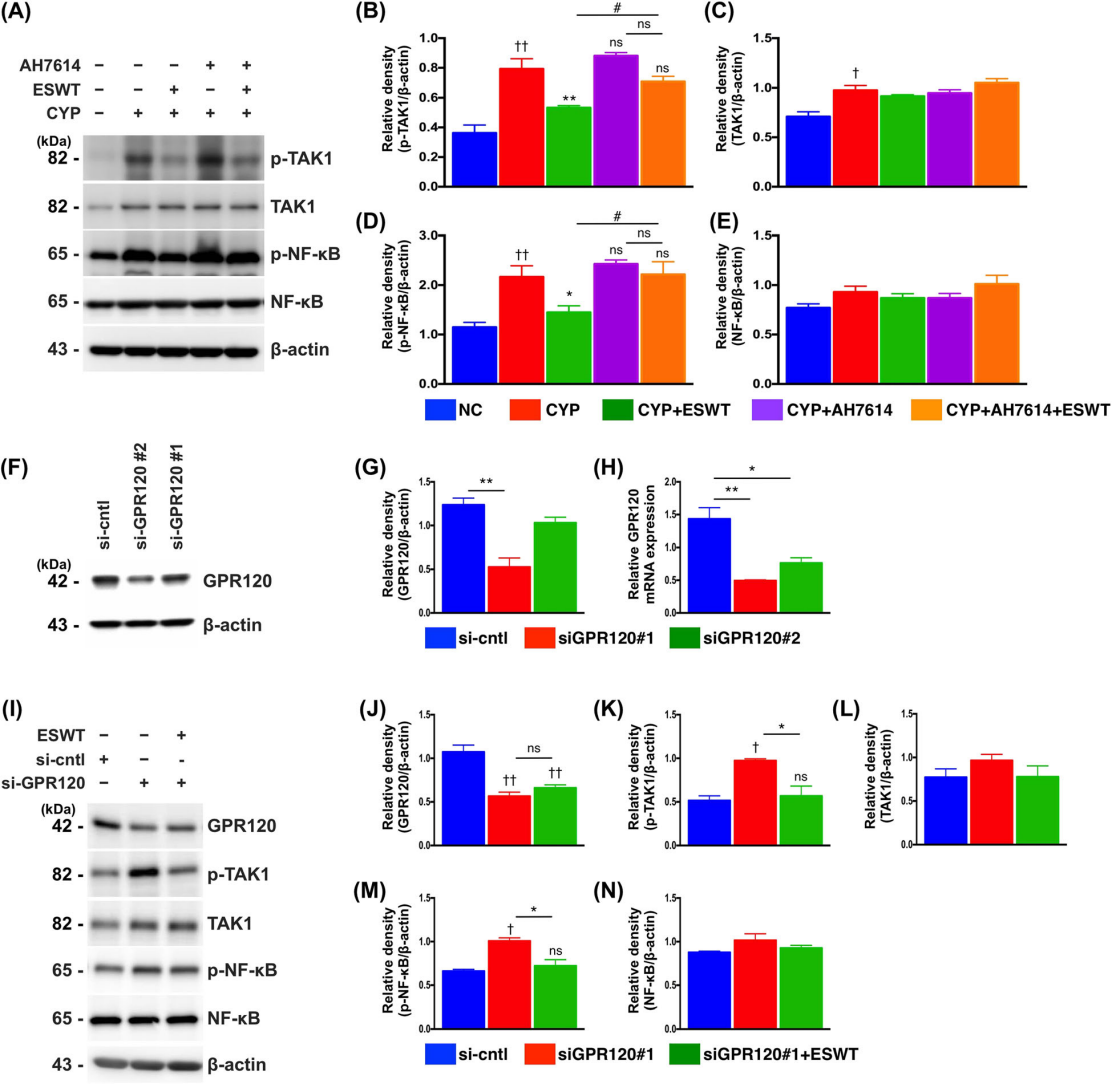
Effect of extracorporeal shock wave treatment (ESWT) and GPR120 antagonist on cyclophosphamide (CYP)-stimulated inflammatory response in urothelial RT4 cells. **a-e** Protein expressions of active pro-inflammatory markers (i.e., p-TAK1 and p-NF-κB) in CYP-stimulated RT4 cells at 4 h with and without prior treatment with GPR120 antagonist (i.e., AH7614) compared to those induced through ESWT. **f-h** Immunoblotting of RT4 cells treated with GPR120-specific or control (si-cntl) siRNA for 48 h showing that exposure to GPR120-specific siRNA reduced endogenous GPR120 mRNA and protein expression by approximately 50%. **i-n** Knockdown of GPR120 significantly impaired the ability of ESWT to reduce p-TAK1 and p-NF-κB. β-actin used as internal control for immunoblotting. Values expressed as the mean ± SD of three independent experiments. *p < 0.05, **p < 0.01 vs. CYP/siGPR120; ^†^p < 0.05, ^††^p < 0.01 vs. control; ^#^p < 0.05, vs. CYP + ESWT; ns, non-significance. Significance of differences determined by one-way ANOVA followed by Bonferroni’s post-hoc comparisons tests

To verify the role of GPR120 in ESWT-induced anti-inflammatory effect, the expressions of phosphorylated TAK1 and NF-κB were determined in AH7614 (i.e., GPR120 antagonist)-pretreated RT4 cells (Fig. 3a-e). The results showed that, in the absence of ESWT, CYP pretreatment induced elevated expressions of phosphorylated TAK1 and NF-κB compared to those at baseline. Addition of AH7614 further increased the expressions of phosphorylated TAK1 and NF-κB. On the other hand, ESWT significantly suppressed the expressions of phosphorylated TAK1 and NF-κB in CYP-pretreated RT4 cells in the absence of AH7614, while the suppressive effects significantly diminished when the cells were pretreated with AH7614.

To further specify the role of GPR120 in ESWT-induced anti-inflammation in the present experimental setting, small interfering-GPR120 (si-GPR120) was used for silencing endogenous GPR120 expression. Silencing of GPR120 expression resulted in approximately 50% reduction in GPR120 mRNA and protein expressions (Fig. 3f-h) and significantly upregulated the expressions of phosphorylated TAK1 and NF-κB in the absence of ESWT (Fig. 3i-n). However, the pro-inflammatory effect was abolished after ESWT so that the expressions of phosphorylated-TAK1 and -NF-κB were comparable to those of the control.

### Effect of GPR120 and ESWT on CYP-induced NF-κB translocation

**Fig. 4:**
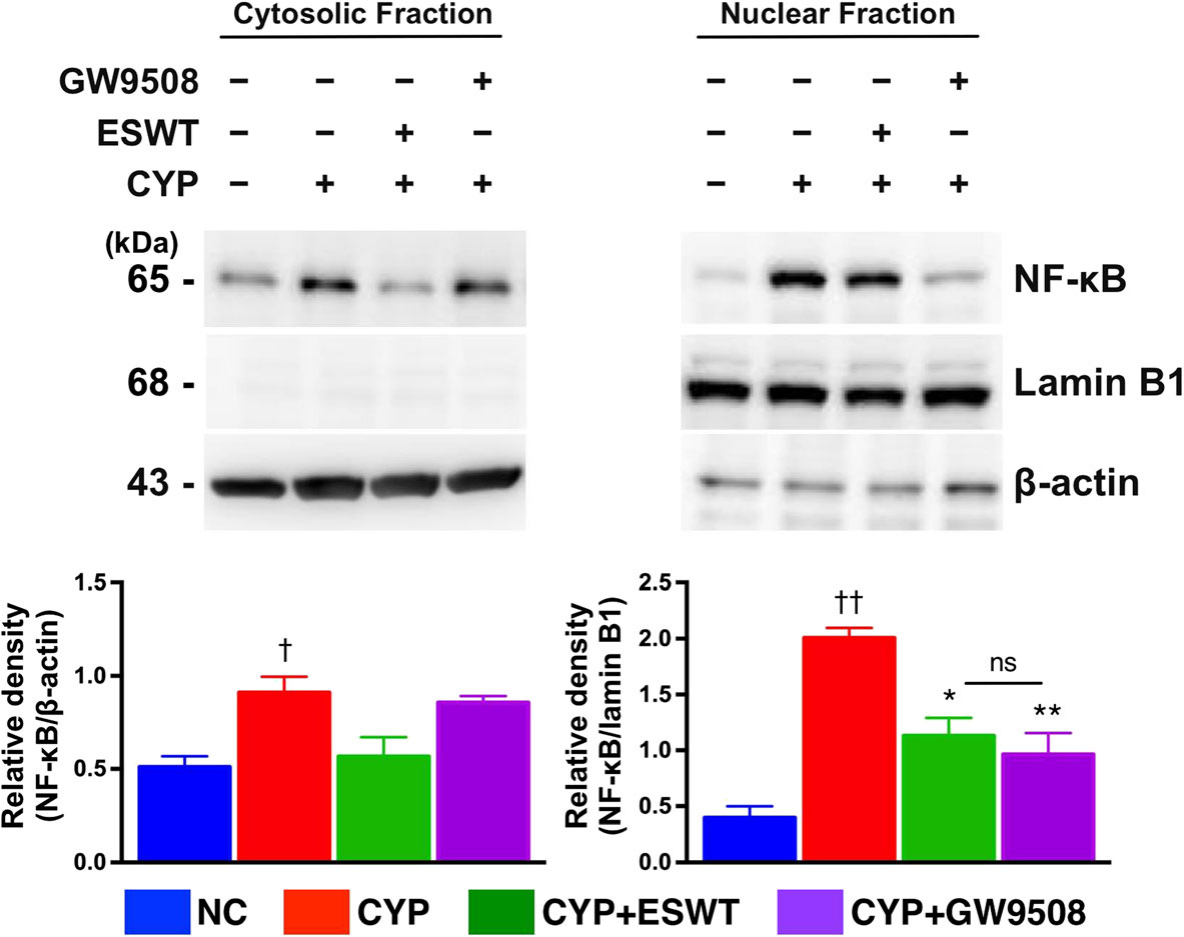
Effects of extracorporeal shock wave treatment (ESWT) and GPR120 agonist on nuclear factor-κB (NF-κB) translocation in cyclophosphamide (CYP)-stimulated RT4 cells. β-actin and lamin B1 used as internal controls for cytosolic and nuclear fractions, respectively. Values expressed as the mean ± SD of three independent experiments. *p < 0.05, **p < 0.01 vs. CYP; ^†^*p* < 0.05, ^††^*p* < 0.01 vs. control; ns, non-significance. Significance of differences determined by one-way ANOVA followed by Bonferroni’s post-hoc comparisons tests

To further characterize the mechanisms through which GPR120 inhibits pro-inflammatory responses, we investigated whether GPR120 prevents the translocation of the p65 subunit of NF-κB to the nucleus. Following exposure to CYP, immunoblotting analysis showed markedly increased NF-κB p65 expression in the nucleus (Fig. 4), which was suppressed by pre-treatment with ESWT and GW9508.

### Urine amount, and urine level of pro-inflammatory cytokines

**Fig. 5:**
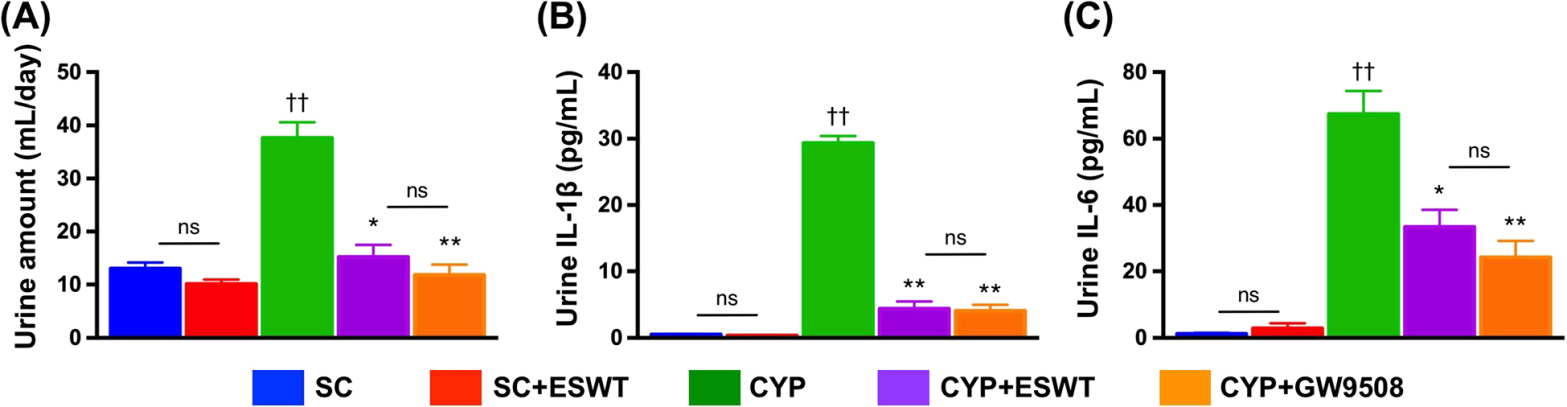
The 24-h urine amount, and urine levels of interleukin (IL)-1β and IL-6 by 72 h in rats following cyclophosphamide (CYP)-induced acute Interstitial cystitis. **a** 24-h urine volume, (**b-c**) Urine levels of IL-1β and IL-6 by the end of 72 h in the five groups of animals (*n* = 6 per group). Values expressed as the mean ± SEM. *p < 0.05, **p < 0.01 vs. CYP; ^†^*p* < 0.05, ^††^*p* < 0.01 vs. controls; ns, non-significance. Significance of differences determined by one-way ANOVA followed by Bonferroni’s post-hoc comparisons tests. SC = sham control; CYP = cyclophosphamide; ESWT = extracorporeal shock wave treatment; GW9508 = GPR120 agonist

CYP-treated rats (150 mg/kg) exhibited increased 24-h urine volume as compared with that in the control rats. By contrast, treatments with ESWT at an energy level of 0.15 mJ/mm^2^ with 300 impulses and GW9508 at the dose of 0.5 μg/kg reduced urine output. Additionally, the pattern of 24-h proteinuria was similar to that of urine volume among the five groups (Fig. 5a). Moreover, compared with that of the control rats, CYP treatment caused significant increases in urine IL-1β and IL-6 concentrations which were reduced following ESWT and GW9508 treatments (Fig. 5b-c).

### Histological evaluation of anti-inflammatory and anti-fibrotic properties of ESWT and GPR120

**Fig. 6:**
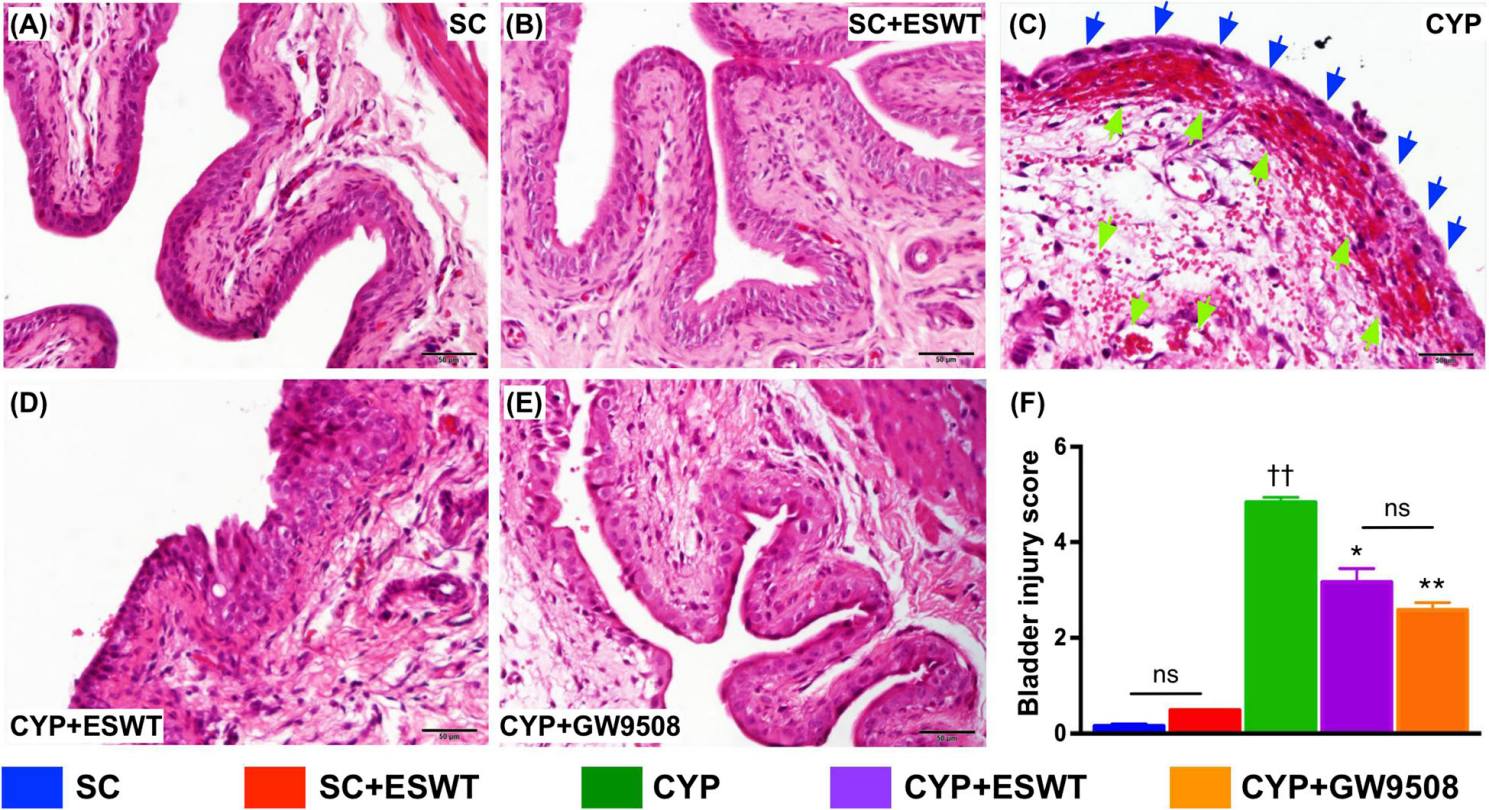
Histological distortion of urinary bladder in rats following cyclophosphamide (CYP) stimulation with and without GPR120 agonist / extracorporeal shock wave treatment (ESWT). Hematoxylin eosin (H&E)-stained bladder sections showing normal urothelium in sham controls with (**a**) and without (**b**) ESWT. Damaged urothelium noted in rats following CYP treatment (**c**), including mucosal thinning. (blue arrows) and edematous lamina propria with inflammatory cell infiltration (green arrows). Significant preservation of urothelial integrity after ESWT (**d**) and GPR120 agonist (i.e., GW9508) (**e**) treatment. **f** Summary of differences in bladder injury score in the five groups of animals (*n* = 6 per group). Values expressed as the mean ± SEM. *p < 0.05, **p < 0.01 vs. CYP; ^††^*p* < 0.01 vs. controls; ns, non-significance. Significance of differences determined by one-way ANOVA followed by Bonferroni’s post-hoc comparisons tests. Scale bars in right lower corner represent 50 μm. SC = sham control; CYP = cyclophosphamide; ESWT = extracorporeal shock wave treatment; GW9508 = GPR120 agonist

**Fig. 7:**
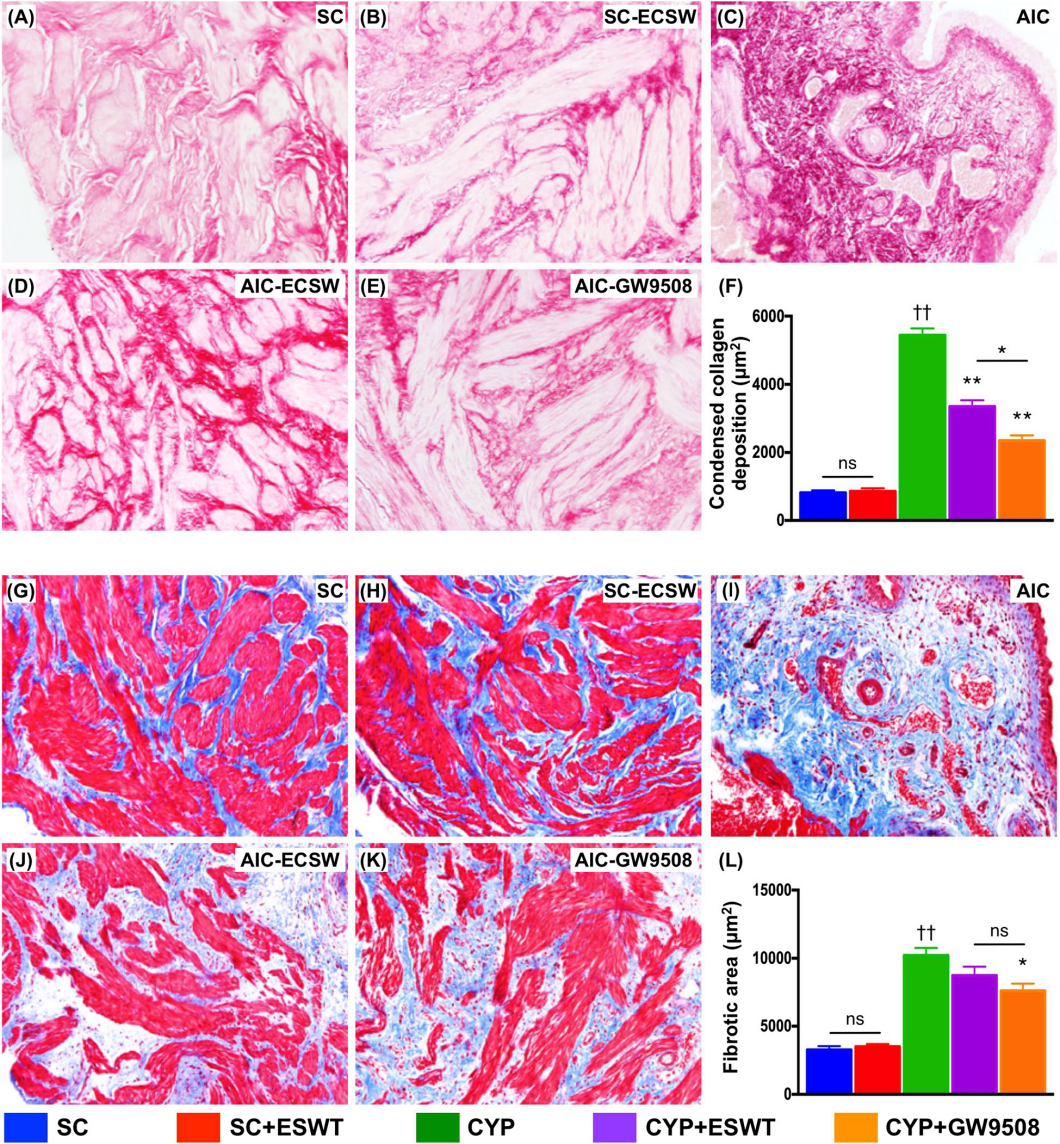
Fibrosis and condensed collagen deposition in rat urinary bladder following cyclophosphamide (CYP) stimulation with and without GPR120 agonist / extracorporeal shock wave treatment (ESWT). Masson’s trichrome and Sirius red-stained bladder sections showing normal urothelium in sham controls with (**a, g**) and without (**b, h**) ESWT. Fibrotic bladder noted in rats following CYP treatment (**c, i**). Significant inhibition of bladder fibrosis after ESWT (**d, j**) and GPR120 agonist (i.e., GW9508) (**e, k**) treatment. **f, l** Summary of differences in bladder fibrotic area in the five groups of animals (*n* = 6 per group). Values expressed as the mean ± SEM. *p < 0.05, **p < 0.01 vs. CYP; ^††^*p* < 0.01 vs. controls; ns, non-significance. Significance of differences determined by one-way ANOVA followed by Bonferroni’s post-hoc comparisons tests. Scale bars in right lower corner represent 50 μm. SC = sham control; CYP = cyclophosphamide; ESWT = extracorporeal shock wave treatment; GW9508 = GPR120 agonist

Compared to the histologically normal bladders in control rats, the urinary bladders of the CYP-treated rats showed notable mucosal thinning and edematous lamina propria with inflammatory cell infiltration (Fig. 6). The histological integrity was significantly preserved after GW9508 or ESWT treatments (Fig. 6). Histological analysis of bladder sections after Masson trichrome and Sirius red staining for studying the degree of fibrosis and collagen deposition, respectively, gave consistent findings compared to those of the H&E-stained sections in all groups (Fig. 7).

**Fig. 8:**
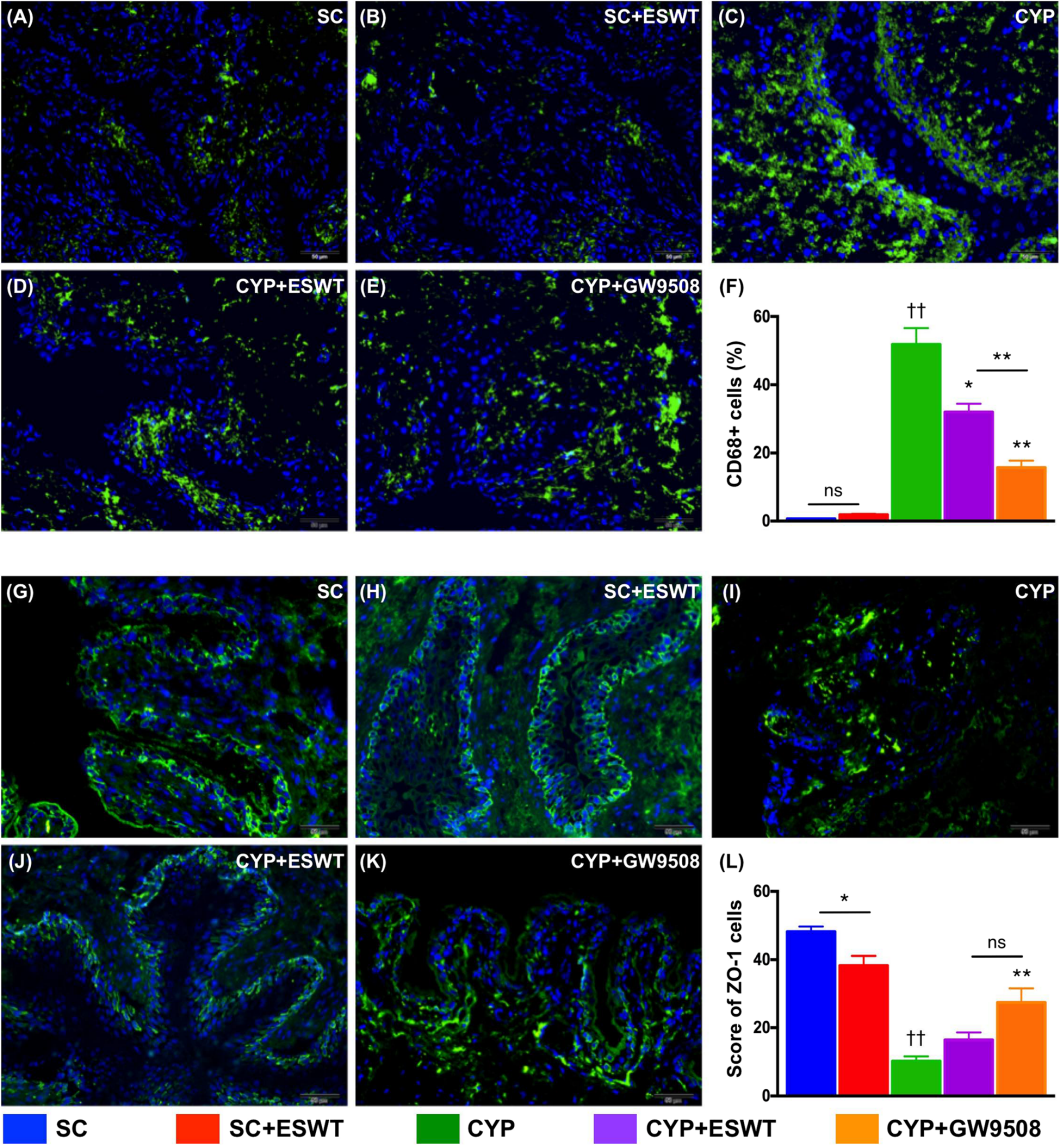
Inflammatory cells infiltration and urothelial tight junction integrity of urinary bladder in rats following cyclophosphamide (CYP) stimulation with and without GPR120 agonist / extracorporeal shock wave treatment (ESWT). Immunofluorescent (IF) staining of bladder sections with CD68 antibody showing normal urothelium in sham controls with (**a**) and without (**b**) ESWT. Inflammation noted in rats following CYP treatment (**c**). Significant attenuation of inflammatory cell infiltration after ESWT (**d**) and GPR120 agonist (i.e., GW9508) (**e**) treatment. **f** Summary of differences in numbers of CD68+ cells in the five groups of animals (*n* = 6 per group). IF staining of ZO-1 showing normal urothelial integrity in sham controls with (**g**) and without (**h**) ESWT. Impaired urothelial integrity noted in rats following CYP treatment (**i**). Significant protection of urothelial integrity after ESWT (**j**) and GPR120 agonist (i.e., GW9508) (**k**) treatment. **f** Summary of differences in numbers of ZO-1+ cells in the five groups of animals (*n* = 6 per group). Values expressed as the mean ± SEM. *p < 0.05, **p < 0.01 vs. CYP; ^††^*p* < 0.01 vs. controls; ns, non-significance. Significance of differences determined by one-way ANOVA followed by Bonferroni’s post-hoc comparisons tests. Scale bars in right lower corner represent 100 μm. SC = sham control; CYP = cyclophosphamide; ESWT = extracorporeal shock wave treatment; GW9508 = GPR120 agonist

Immunofluorescent study revealed a CYP-induced increase in the numbers of CD68+ cells and a decrease in distribution of the tight junction proteins ZO-1 as compared with those in the normal controls. Conversely, both treatments were found to reduce the infiltration of inflammatory CD68+ cells in the urothelium and preserve urothelial integrity of the bladder (Fig. 8).

### Protein expressions of pro-inflammatory markers after ESWT and GPR120 treatments

**Fig. 9:**
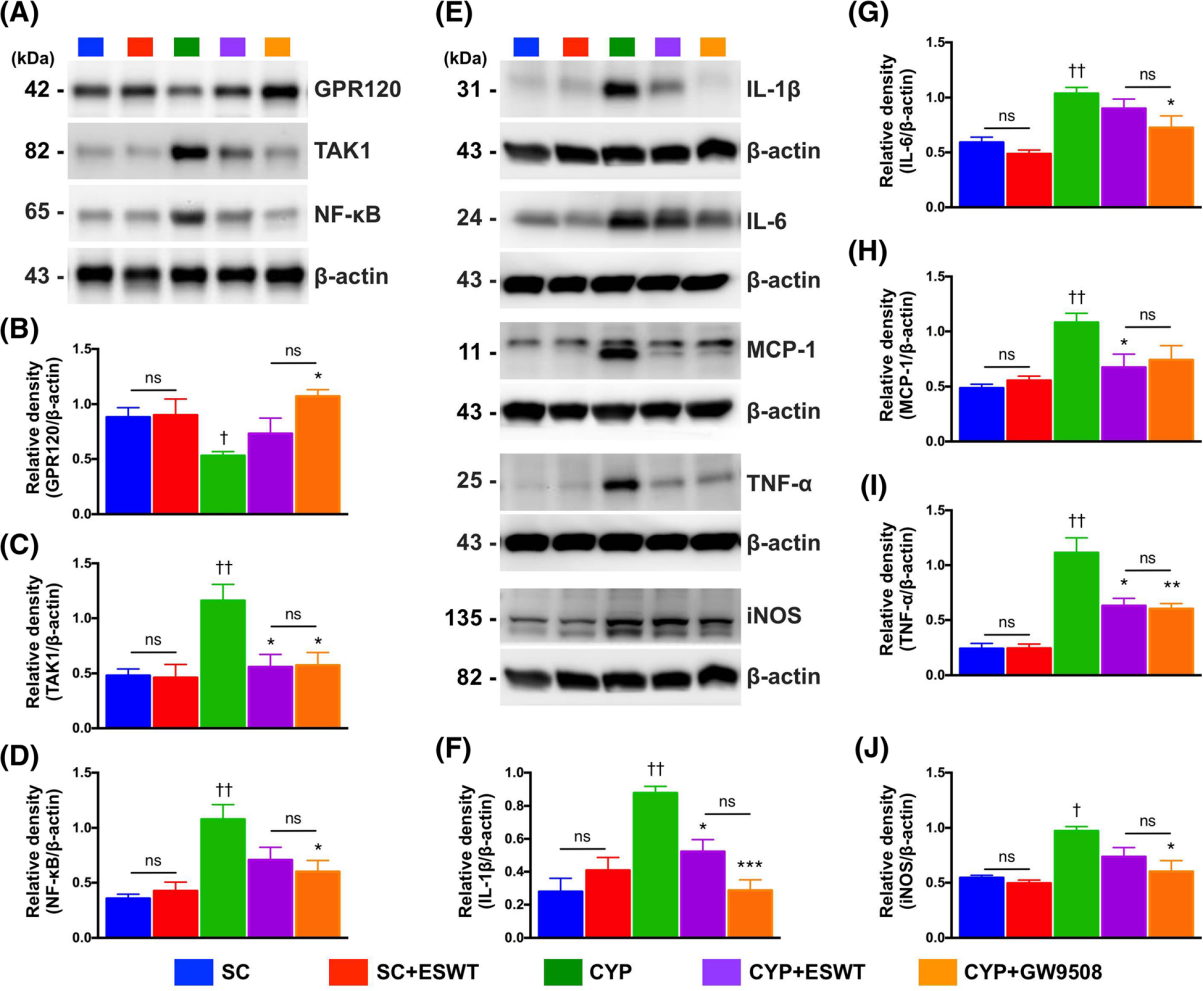
Expressions of downstream molecules of GPR120- and NF-κB-signaling pathway in rat urinary bladders following cyclophosphamide (CYP) stimulation with and without GPR120 agonist / extracorporeal shock wave treatment (ESWT). **a-d** Protein expressions of pro-inflammatory (i.e., TAK1, NF-κB) and anti-inflammatory (i.e., GPR120) markers. **e-j** Protein expressions of IL-1β, IL-6, MCP-1, TNF-α, and iNOS (i.e., downstream molecules of NF-κB mediated-inflammatory signaling pathway) in CYP-treated rat bladder with and without GPR120 agonist / extracorporeal shock wave treatment (ESWT) (*n* = 6 per group). Values expressed as the mean ± SEM. *p < 0.05, **p < 0.01 vs. CYP; ^†^*p* < 0.05, ^††^*p* < 0.01 vs. controls; Significance of differences determined by one-way ANOVA followed by Bonferroni’s post-hoc comparisons tests. SC = sham control; CYP = cyclophosphamide; ESWT = extracorporeal shock wave treatment; GW9508 = GPR120 agonist

CYP-induced bladder inflammation, as reflected in significantly reduced GPR120 and increased TAK1 and NF-κB protein expressions, was significantly suppressed after ESWT treatment as shown in the restoration of TAK1/NF-κB, and GPR120 expressions in the urinary bladder (Fig. 9a-d). Treatment with CYP also led to significant increase in expressions of NF-κB-targeted pro-inflammatory mediators compared to those in the control group. ESWT treatment also significantly suppressed the protein expressions of IL-1β, IL-6, MCP-1, TNF-α and iNOS compared to those in the CYP-treated group. Similarly, GW9508 treatment notably alleviated CYP-elicited bladder inflammation (Fig. 9i).

## Discussion

The current study, which investigated the underlying mechanisms of ESWT treatment in acute IC rat, provided several valuable implications. First, our results showed that GPR120 functioned as an ESWT receptor/sensor in urothelial RT4 cells and urinary bladder. Second, activation of GPR120 by ESWT, DHA ω-3 FAs (natural GPR120 ligands), and GW9508 (GPR120 synthetic agonist) was found to be anti-inflammatory through the inhibition of TAK1 (i.e., an activator of NF-κB), which blocked the downstream NF-κB signaling. Finally, GPR120 activation by ESWT and GW9508 restored bladder uroepithelial integrity and suppressed the inflammatory response at the cellular and protein levels.

The precise etiology of IC, a chronic bladder condition with common symptoms of urinary urgency, frequency, nocturia and pelvic pain (Theoharides et al. 2001), is still uncertain. It is suggested that damage to the bladder surface glycosaminoglycans layer disrupts its protective barrier function, leading to increased urothelial permeability of the and bladder and giving rise to inflammation and pain. (Gonzalez et al. 2014; Chen et al. 2014a, b; Theoharides et al. 2001; Hurst et al. 1996; Hughes Jr. et al. 2014; Parsons 1996; Ha and Xu 2017; Grover et al. 2011). Recent research suggested that consumption of ω-3 EPA and ω-3 DHA may suppress urological inflammation through GPR120 activation (Tamma et al. 2015).

Our previous findings have demonstrated that ESWT significantly attenuated CYP-induced acute IC in rat though inhibiting inflammation and oxidative stress both in vitro and in vivo (Chen et al. 2014a). In addition, combined therapy with melatonin and ESWT was superior to either treatment alone in protecting against CYP-induced acute IC [Chiang HJ, 2014; 2349–4425 (Online)]. On the other hand, the exact physiological mechanism by which ESWT ameliorates IC-associated inflammation is incompletely understood. To the best of our knowledge, this is the first study to demonstrate that ESWT treatment ameliorated CYP-induced inflammatory reactions in an experimental setting of IC through GPR120 activation. Significant intergroup differences were noted for GPR120, TAK1, NF-κB and NF-κB-targeted inflammatory molecules. ESWT-induced GPR120 upregulation was found to reduce immune cell infiltration and preserve urothelial integrity. Moreover, our results with GW9508 (GPR120 agonist) are in agreement with those using ESWT in the current experimental model. Furthermore, Moayednia et al. demonstrated the efficacy of ESWT in treating chronic pelvic pain syndrome with short-term follow-up up to12 weeks (Moayednia et al. 2014). Feasibility of the clinical use of ESWT for treating IC remains to be elucidated.

Oral medications and intravesical drug instillations are currently the most popular therapies in routine clinical practice (Ha and Xu 2017). The downside of intravesical treatment is that drug delivery requires painful urethral and vesicular instrumentation with the potential risk of urinary tract infection (Ha and Xu 2017; Lasdun et al. 1989). The potential solution is the development of non-invasive therapeutic approaches.

## Conclusion

**Fig. 10:**
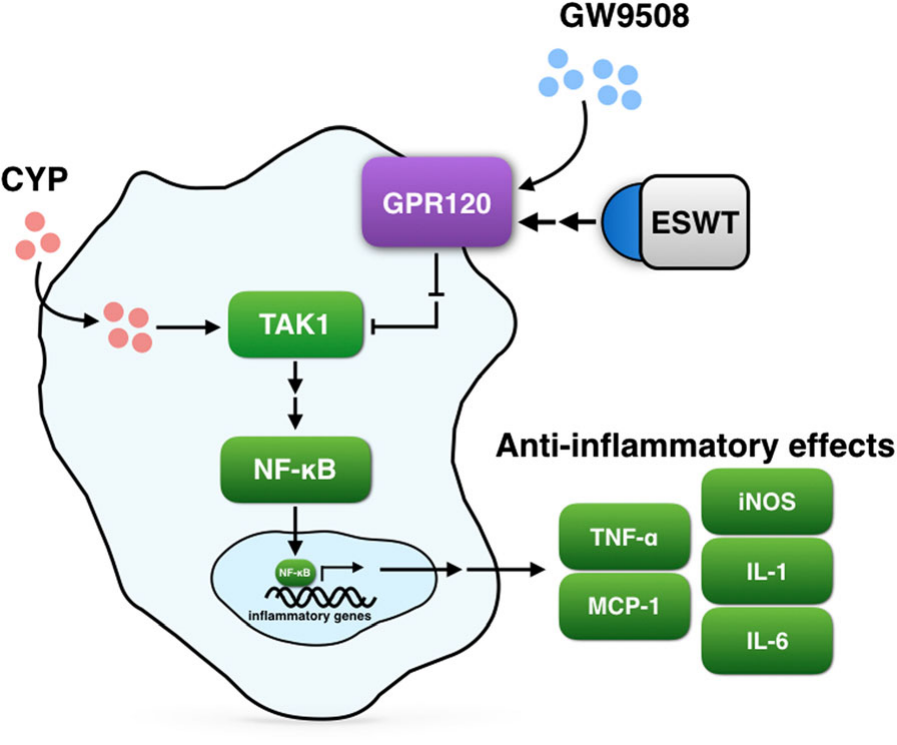
Anti-inflammatory effects mediated by G-protein coupled receptor 120 (GPR120) in an experimental setting of interstitial cystitis. Stimulation of GPR120 through GPR120 agonist (i.e., GW9508) and extracorporeal shock wave treatment (ESWT) causing inhibition of transforming growth factor beta-activated kinase 1 (TAK1) and nuclear factor transcription factor (NF-κB), resulting in suppressed expressions of tumor necrosis factor-α (TNF-α), monocyte chemoattractant protein-1 (MCP-1), iNOS = inducible nitric oxide synthase as well as interleukin (IL) 1 and 6. Protein-protein interaction: activation or interaction (→); inhibition (⇥), indirect effect (→→)

The present preclinical study demonstrated that minimally invasive ESWT substantially suppressed bladder inflammation and preserved urothelial integrity. The findings of the present study, therefore, raise the possibility of combined clinical use of ESWT and omega-3 FAs supplement for treating patients with IC refractory to conventional therapy. The proposed mechanisms of ESWT underlying the observed improvement in the outcome of acute IC based on the findings of the current study are summarized in Fig. 10.

### Study limitations

The present study has its limitations. First, the use of CYP for creating an animal model of acute IC may not reflect the clinical condition of IC which is a more chronic situation. Since the treatment course was only 3 days in the current experimental setting, the therapeutic effects of long-term treatment remain unclear. Second, urodynamic study, which is an effective tool for functional assessment of the bladder, was not performed in the present model so that the functional improvement after treatment warrants further elucidation. Third, the current study did not include another group of animals treated with AH7614 to show the abolishment of ESWT-induced anti-inflammatory effect on urothelium in vivo. Fourth, since CYP requires metabolic breakdown to acrolein in liver and kidney to produce its adverse effects, the use of CYP instead of acrolien in the in vitro experiments of the present study may raise the concern of direct action of the tested drugs on cellular CYP metabolism (e.g., inhibition) rather than their effects on GPR120. Finally, although urinary tract infection was unlikely within a three-day experiment period, it was a potential confounder that cannot be ruled out since urinalysis was not routinely performed. In conclusion, the results of the current study demonstrated that GPR120 upregulation may be one of the mechanisms by which ESWT exerts its anti-inflammatory action, which was found to be effective for the treatment of acute IC in a rodent experimental model.

## Abbreviations

CYP: Cyclophosphamide
DHA: Docosahexaenoic acid
ESWT: Extracorporeal shockwave treatment
GPCRs: G protein-coupled receptors
H&E: Hematoxylin and eosin
IC: Interstitial cystitis
IL: Interleukin
iNOS: Inducible nitric oxide synthase
MAPKs: Mitogen-activated protein kinases
MCP-1: Monocyte chemoattractant protein-1
NF-κB: Nuclear factor transcription factor (NF-κB)
SC: Sham-operated rats
siRNA: Small interfering RNA
TAB1: TAK1 binding protein
TAK1: TGF-β activated kinase 1
TNF-α: tumor necrosis factor-α
ω-3 FAs: Omega-3 fatty acids
